# Cell-Based Therapies for Joint Disease in Veterinary Medicine: What We Have Learned and What We Need to Know

**DOI:** 10.3389/fvets.2018.00070

**Published:** 2018-04-16

**Authors:** Sophie Helen Bogers

**Affiliations:** Department of Large Animal Clinical Sciences, Virginia-Maryland College of Veterinary Medicine, Blacksburg, VA, United States

**Keywords:** mesenchymal stem cells, osteoarthritis, platelet-rich plasma, autologous-conditioned serum, cell-based therapies, Food and Drug Administration regulation, autologous conditioned plasma

## Abstract

Biological cell-based therapies for the treatment of joint disease in veterinary patients include autologous-conditioned serum, platelet-rich plasma, and expanded or non-expanded mesenchymal stem cell products. This narrative review outlines the processing and known mechanism of action of these therapies and reviews current preclinical and clinical efficacy in joint disease in the context of the processing type and study design. The significance of variation for biological activity and consequently regulatory approval is also discussed. There is significant variation in study outcomes for canine and equine cell-based products derived from whole blood or stem cell sources such as adipose and bone marrow. Variation can be attributed to altering bio-composition due to factors including preparation technique and source. In addition, study design factors like selection of cases with early vs. late stage osteoarthritis (OA), or with intra-articular soft tissue injury, influence outcome variation. In this under-regulated field, variation raises concerns for product safety, consistency, and efficacy. Cell-based therapies used for OA meet the Food and Drug Administration’s (FDA’s) definition of a drug; however, researchers must consider their approach to veterinary cell-based research to meet future regulatory demands. This review explains the USA’s FDA guidelines as an example pathway for cell-based therapies to demonstrate safety, effectiveness, and manufacturing consistency. An understanding of the variation in production consistency, effectiveness, and regulatory concerns is essential for practitioners and researchers to determine what products are indicated for the treatment of joint disease and tactics to improve the quality of future research.

## Introduction

Regenerative medicine focuses on therapies that regrow, repair, or replace damaged cells or organs (1). Cell-based therapies derived from tissues such as blood, bone marrow, and adipose tissue are a cornerstone of regenerative medicine. These products contain enhanced quantities of biological response modifiers, which are normally produced in the body at low levels and include stem cells, anti-inflammatory cytokines, growth factors, or a combination (2–4). The potential to both relieve symptoms of disease and repair damaged tissue have led to investigation of cell-based therapy for a wide range of human and animal orthopedic disease. As of February 2018, the U.S. National Institutes of Health had 57 active or recruiting clinical trials investigating cell-based therapies for osteoarthritis (OA) alone (5).

Osteoarthritis is an irreversible, complex disease that involves all tissues of the joint in a cycle of inflammation and tissue degradation (6). OA affects over 80% of horses >15 years of age and up to 2/3 of Thoroughbred racehorses, making it one of the highest causes of wastage and loss of use in this population (7, 8). Treatment in these populations has traditionally been intra-articular corticosteroid therapy, supplemented with polysulfated glycosaminoglycans, glucosamine and chondroitin sulfate, or hyaluronic acid (9). However, traditional pharmacological therapies decrease symptoms as opposed to modifying or reversing the disease process. Although some pharmaceuticals have been classified as disease-modifying osteoarthritic drugs (DMOADs) on initial clinical and preclinical trials, subsequent meta-analysis has shown insufficient levels of disease-modifying effects in humans (10). As a result, optimism is high for cell-based therapies that alter the inflammatory cycle of the disease, regenerate damaged tissues or, ideally, both.

Veterinary medicine’s commercial environment and the perceived benefits of cell-based therapies as delivering disease-modifying and reparative effects, as well as pharmaceutical restrictions in equine athletes (11–13), has led to widespread use of cell-based therapies in horses and dogs for OA Table 1. Commonly used cell-based products include autologous-conditioned serum (ACS), platelet-rich plasma (PRP), and expanded or non-expanded mesenchymal stem cell (MSC) products. Quality of published literature and practitioner understanding about safety, efficacy, and consistency of these products varies. Due to funding constraints, many studies have a low number of animals, a lack of control groups or have not progressed beyond pilot data. In addition, variability derived from factors including individual donor and processing method challenges our ability to draw meaningful conclusions (14–20).

**Table 1:** Regenerative medicine products used in the dog and horse for OA.

Category	Description	Examples of US based veterinary suppliers/products	Effects in OA
Autologous-conditioned serum	Autologous blood product that increases anti-inflammatory cytokines including interleukin-1 receptor antagonist	IRAP (Dechra/Orthokine); IRAP II (Arthrex); MediVet; Biologics; EC-ACS (Vetlinebio)	Improved lameness, synovial thickness, and cartilage fibrillation (21, 22)
Platelet-rich plasma (PRP)	Autologous blood product that contains growth factors including IGF-1 and PDGF	MediVet; VetStem; Osteokine (Dechra); Arthrex ACP; V-Pet (Pall Life Sciences); PRPKits.com; DrPRP USA; RegenKit-BCT (RegenLab); E-Pet (V-Care); V-PET (Nupsala)	Variable response to intra-articular injection in horses, some show reduction in lameness and joint effusion (23–25). In dogs has a pain-relieving effect that is slower onset but similar effect compared with corticosteroid injection (26, 27)
Autologous protein solution	Autologous blood product that contains both growth factors and ant-inflammatory cytokines via a 2 step process	Pro-Stride; N-Stride	Reduced clinical signs of pain and lameness in dogs at 12 weeks (28) and horses at 14 days and 12 weeks *via* client assessment (29)
Adipose-derived stromal vascular fraction	Digest of autologous adipose tissue that contains ~1–2% of CFU-fibroblasts	VetStem (Biopharma)	Subjectively less effective than cultured bone marrow-derived stem cells when compared with placebo for experimental OA in horses (30). Functional improvements in naturally occurring and induced canine OA, with some evidence of improvement when paired with PRP (31, 32)
Mesenchymal stem cells (MSCs)	Autologous or allogeneic plastic adherent cells that are commonly isolated from bone marrow or fat. Capable of differentiating into osteogenic, chondrogenic, or adipogenic cell lines	Variable—stem cell therapy may be offered by comparative orthopedic research laboratories	Bone marrow-derived MSCs showed no significant effects for naturally occurring OA; however, it can improve return to work of horses with intra-articular soft tissue injury (33). Canine studies using adipose-derived MSCs show improved functional outcomes, their effect may be complemented when PRP is used as a vehicle (34)

Researchers, practitioners, and regulatory agencies are understanding that collective and regulated data collection will help to overcome challenges associated with product variability and study limitations (35). Guidelines set in the USA by the Food and Drug Administration (FDA) are an example of how government-led regulation could force the industry to prove product efficacy, manufacturing quality, and safety before commercialization. The guidelines are controversial given inevitability that following this process will slow, or even stall, the transition of cell-based products from experimental to clinical use. However, the impact on research quality and informed practitioner use will no-doubt drive forward development of OA cell-based therapies that do meet current optimism. Until that time, we can only assess the efficacy of biological therapies used in equine and canine OA in light of the disease environment, product variation, and legislative recommendations. Understanding clinical and experimental findings in light of these elements is essential for practitioners and researchers to determine what products could be indicated for treatment of joint disease and to highlight areas of future research.

## Cell-Based Therapies Currently Used in Dogs and Horses for Joint Disease

Cell-based therapies investigated in horses and dogs include blood-derived products such as ACS, autologous protein solution (APS), and PRP, as well as products containing MSCs such as adipose-derived stromal fraction, bone marrow aspirate concentrate (BMAC), cultured adipose-derived stem cells [adipose-derived MSC (AdMSC)], and cultured bone marrow-derived stem cells (BMSCs). All cell-based products are multimodal, containing multiple and combinations of growth factors, cytokines, and cells. The combination of factors may trigger an anabolic, chemotactic, inflammatory, anti-inflammatory, or immune-mediated response. This review describes how the complex nature of the composition and biological effect of cell-based products is further influenced by product source, donor variation, preparation technique, storage, injection vehicle, and characteristics of the disease environment in our veterinary patients.

Inherent variation in cell-based products and variation in study size and quality in veterinary species leaves veterinary researchers and practitioners to piece together currently available species-specific evidence, or reference human literature, to make clinically relevant decisions. Due to a lack of robust clinical data, *in vitro* data that highlight mechanisms of action cannot be overlooked. In addition, there are few studies that directly compare different cell-based therapies for OA (30, 36–38), and foundational information such as optimum processing and storage methods, as well as safe and effective doses and dosing regimens, are inconclusive (21, 22, 39, 40). Therefore, critical analysis of available information as well as a thorough understanding of key therapeutic elements of cell-based therapies is essential for the practitioner and researcher alike.

## MSC Products

**Figure 1:**
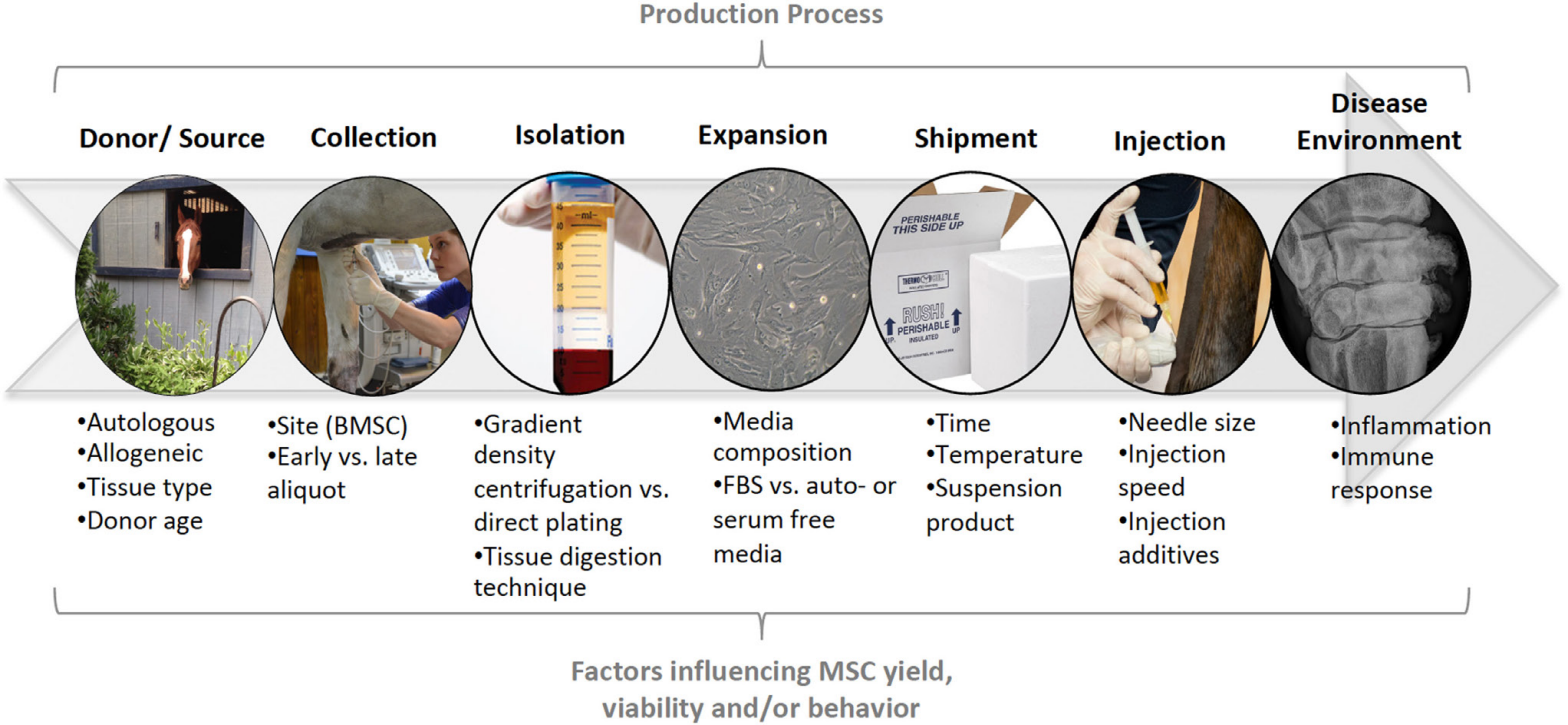
Summary of the production process for mesenchymal stem cells (MSCs). At each stage in the production process (top), specific factors may influence the yield, viability, and/or behavior of MSCs in veterinary patients. These factors need to be considered during clinical use of MSCs and controlled for, or described, in preclinical and clinical research.

Stem cells are adult or embryonic in origin. Adult stem cells do not exhibit telomerase activity, a marker of stem cell self-renewal in embryonic stem cells, so undergo senescence in 30–40 population doublings. However, this gives them clinical advantages such as reduced tumorogenicity when used *in vivo* (41, 42). MSCs have regenerative, anti-inflammatory, immunomodulatory, and trophic functions (43). As a result of the multifaceted nature of stem cell function, they are being investigated in the treatment of a wide range of diseases, with promise to aid in cartilage regeneration as well as amelioration of inflammation during OA. The beneficial effects of MSCs for intra-articular soft tissue injury was first demonstrated by Murphy et al. (44), in a caprine menisectomy model of OA. There was regeneration of the medial meniscus, which subsequently ameliorated OA development. Since that time, MSCs have been used successfully for the treatment of intra-articular soft tissue injury in horses and dogs (45, 46), as well as for cartilage regeneration (47, 48). However, there have been more variable outcomes when used for primary OA (30, 49). As with other cell-based therapies, there are many sources of variation that need to be considered before treatment of patients with intra-articular MSCs, or before conducting or reviewing clinical or preclinical research. Such factors include stem cell source, collection, and propagation techniques, effects of shipping and transportation, as well as what vehicle and what needle size will be used for injection Figure 1.

### Considerations for Collection, Propagation, and Administration of MSCs

Bone marrow and adipose tissue are the most common tissues harvested for MSCs in veterinary species. These tissues are either processed as a point-of-care product or shipped to a laboratory to process the tissue and expand MSCs in culture for 2–4 weeks before shipment back to the end-user. Point-of-care products can be used within hours of tissue collection. BMAC is bone marrow aspirate that is collected with an anti-coagulant then gradient-density centrifugation removes red blood cells, granulocytes, immature myeloid precursors, and platelets. The resulting cells are mononuclear cells, comprising approximately 13% of the total nucleated cells in horses (50). Within this number, there are MSCs, which have been found to be approximately 0.001–0.01% of mononuclear cells (51). Adipose-derived stromal vascular fraction is a similar gradient-density centrifugation product derived from adipose tissue. Like BMAC, there are low numbers of CFU fibroblasts, the MSC fraction proposed to be active in the product, for example, canine point-of-care adipose preparations are reported to contain 1.72% of CFU fibroblasts (52, 53). The low fraction of MSCs are a point of controversy in these products, with some arguing that marketing products as a stem cell source is misleading. However, proponents of the products suggest that favorable clinical results may be related to paracrine or immunomodulatory effects rather than providing a direct MSC source. These arguments are supported by findings that freshly isolated cells from equine BMAC do not undergo trilineage differentiation (54), but do induce endogenous MSC proliferation, chemotaxis, and paracrine response (55). Differences between therapeutic activity of CFU-fibroblasts and other cytokines or growth factors in point-of-care products and expanded MSCs, as well as effective or bio-equivalent doses of each are currently unknown.

Mesenchymal stem cells are harvested from the end-user (autologous) or harvested from one animal and used in another of the same species (allogeneic). Veterinary medicine most commonly uses autologous MSCs because the perceived risk of immune rejection is lower. However, findings that allogeneic MSCs can decrease proliferation of T cells, alter the phenotype of macrophages, and cause reduction in inflammatory cytokines horses in a similar manner to autologous MSCs challenge this perception (56–59). Allogeneic MSCs are desirable because they can be used “off the shelf” as opposed to waiting for MSCs to be expanded from autologous tissue. For this reason, allogeneic adipose- and bone marrow-derived stem cells are available for horses and dogs in Australia (60), and recently Harman et al. (61) completed an FDA-registered clinical trial for the treatment of canine OA with allogeneic AdMSCs. However, several smaller *in vivo* studies in horses have found transient inflammation lasting 24–48 h after allogeneic MSCs have been injected into joints (36, 62–64). Similar transient inflammation may be elicited with autologous MSCs on the initial injection (58, 62). A limitation of studies that investigate single intra-articular doses of allogeneic MSCs in a small group of horses or dogs is that they may give a false representation of the safety of these products. *In vitro* studies show that certain inflammatory conditions cause equine MSCs to express MHC II, which induces immune detection in unmatched recipients (65, 66). Such alloantibody production is induced after intravenous, intraocular, and intraarterial injection of allogeneic BMSCs and AdMSCs in horses (67). It is unknown if the alloantibody response to intra-articular injection is similar; however, repeat intra-articular injection 4 weeks after treatment with the same allogeneic cell line increased synovial fluid cell counts, total protein, and lameness in horses for 24 h (64). These findings indicate that even if authors such as Harman et al. (61) conduct studies in a prospective, controlled and double-blinded manner, if measurements are taken after the acute period and are based on a single injection, safety of these products may be falsely represented. Specifically, the results cannot be extrapolated to repeat use of allogeneic cell lines and missing evaluation in the initial 24–48 h would fail to detect initial transient inflammation or pain.

Bone marrow-derived MSCs and AdMSCs are the most common culture-expanded MSCs used in veterinary patients, with BMSCs dominating equine and AdMSCs dominating canine veterinary medicine. In horses, BMSCs are harvested from the sternum or ileum with a 10- to 11-G Jamshidi needle (68–70). Although cardiac puncture is a potential danger with sternal collection, harvest from the 5th sternebra avoids iatrogenic trauma to the apex of the heart and the harvest site can be confirmed with ultrasound (69). Neither MSC viability, density, nor proliferation are different between bone marrow aspirates obtained from the sternum or ileum of young horses (2–5 years old) (71); however, in middle-aged horses (13 years old), sternal samples have a greater density of MSCs than ileal samples (72). Therefore, the sternum is most commonly chosen as the harvest site in middle-aged to older horses. In addition, the highest yield of cells occurs in the initial 5 mL collected, so collection of large volumes of bone marrow is not necessary (71). A higher yield of cells can be achieved by advancing the needle into the sternum 5 mm three times to harvest from four sites, rather than harvesting from one site (73). However, the benefit of this technique is negated after the first passage in culture, so the technique is most relevant for point-of-care preparations. AdMSCs are collected from adjacent to the tail head in horses (74) and harvest from the falciform ligament eases collection in dogs compared with harvest from peritoneal fat (75). The same sites for tissue collection are used for point-of-care systems.

Once collected, adipose or bone marrow tissue samples are shipped to a laboratory for expansion in culture. Laboratories vary slightly in methods of isolation and culture of MSCs; however, strict aseptic technique as well as quality control measures increase safety and consistency of the product (35). In addition, demonstrating Good Manufacturing Practice will be an essential part of ensuring quality and consistency of products seeking regulatory approval (35, 76, 77). In general, bone marrow-derived mononuclear cells are isolated from bone marrow aspirate *via* gradient-density centrifugation as described for BMAC, then plated onto adherent plastic where they undergo population doublings until there are a sufficient number of cells (68, 78, 79). An alternative approach is to transfer neat bone marrow into adherent plastic tissue culture flasks and culture with growth medium with the disadvantage of reducing the density of colony-forming units (54). Both techniques rely on the inherent property of MSCs to adhere to plastic (80). Adipose tissue is mechanically and enzymatically digested before centrifugation to separate the cellular fraction from the adipose fraction before expansion in culture. Comparison of equine AdMSC and BMSC cultures shows that AdMSCs are able to undergo more population doublings (81, 82), which has also been described in humans (83).

In veterinary species, MSCs are often expanded using cell culture media containing fetal bovine serum (FBS), which raises concerns for immunogenicity, consistency in bio-composition from batch to batch and downstream effects on MSC function. FBS proteins cause antibody production in humans, despite washing MSCs before implantation (84) and 89% of horses had antibody production after systemic injection of MSCs (67). Xenoproteins may cause adverse effects upon repeat injection, even if the MSCs are autologous. For example, equine BMSCs cultured in FBS caused an inflammatory reaction upon repeat intra-articular injection, whereas BMSCs cultured for 2 days in serum-free media did not (64). In an attempt to overcome antigenicity of xenoproteins, some laboratories culture MSCs in serum-free media, autologous platelet lysate, or autologous serum for at least 48 h before harvest. Of these options, serum-free media has the most consistent bio-composition, given the wide variation in growth factors and cytokines described for blood-derived products (refer to the next section). However, Clark et al. (85) found that serum-free media can cause alterations in the ability of equine BMSCs and canine AdMSCs to cause an immunomodulatory response, which may affect the therapeutic efficacy of serum-free cultured MSCs. Immunomodulatory properties of canine and equine MSCs cultured in platelet lysate or autologous serum have not been investigated; however, media containing platelet lysate or FBS caused similar proliferation and viability for equine umbilical cord, bone marrow, and adipose-derived MSCs if the additives did not exceed 30% of the culture media (86, 87). A caveat is that platelet lysate media has reduced ability to isolate cells and a tendency to induce adipogenesis after 4 days so short-term use is recommended (86). Despite current clinical use of media additives from platelet lysate, to autologous serum, to serum-free culture, the effects of media additives on MSC function are largely unknown for veterinary species. Given large variation in cytokines and growth factors from equine and canine blood-derived products, it is likely that additives have varying effects on MSCs with unknown effects on their eventual therapeutic efficacy (14–20).

Following culture, expanded MSCs are shipped from a laboratory to the end-user. However, the shipping time, temperature, and suspension product can influence cell viability (39, 88). Although there are not enough studies to draw a consensus about the best protocol for shipping equine and canine MSCs, time of transport limits MSC viability so administration should be within 12–24 h of cell harvest (39, 40, 88). While one study found cell mortality of 30–40% after 12 h (39), other studies have found 8–10% cell mortality at 24 h (40, 88, 89). Given that a 10% or less reduction in cell viability is found in studies with refrigeration at 4–8°C, this is the most commonly applied shipping condition. For short-term (12–24 h) shipment, no significant effects on cell viability have been found between suspension products (39, 88). However, some blood or bone marrow-derived suspension products rapidly increase cell mortality rates after 24 h (88). In addition, there are conflicting findings when shipping temperatures are directly compared (39, 40). Therefore, laboratories should conduct their own quality control tests to find the best shipping protocols and packaging for their products.

Addition of other intra-articular medications, as well as needle-size selection may influence MSC viability. Just under half (46%) of equine practitioners add adjunctive antimicrobials to intra-articular medications (9). However, addition of high levels of antibiotics such as aminoglycosides, enrofloxacin, and ceftiofur compromises MSC viability (90, 91). Although the antimicrobial concentrations tested were supraphysiological for systemically administered antimicrobials, both gentamicin and amikacin at doses used for intra-articular injection caused >95% equine BMSC death within 2 h *in vitro* (91). In addition, injection through small gauge needles reduces the viability and proliferative potential of equine MSCs (88, 92). In an effort to optimize cell viability, MSCs should not be injected concurrently with antibiotics, and implantation needles 20 G or greater should be used for intra-articular injection. It is unknown if canine MSCs require a different needle gauge for injection. However, injection of human MSCs through 25 G needles did not affect viability, suggesting either species differences or confounding factors such as injection speed or needle length, which may influence sheer stress on cells (93, 94). Some horse and dog clinical studies for OA suspended MSCs in PRP (34, 37, 95). However, suspension of cultured MSCs or point-of-care cell-based products with blood-derived products such as PRP, ACS, or autologous serum adds complexity and variation to joint therapies in veterinary medicine because of potential for products to interact and also because it is unclear what substance is having the primary therapeutic effect. Dahr et al. (96) found alterations of equine BMSC proliferation and differentiation when exposed to PRP, so addition of these substances could affect therapeutic activity of injected MSCs. Both *in vitro* and *in vivo* studies need to be performed to understand the biological effect of combining products before widespread use of specific combinations.

### Stem Cells for Cartilage Resurfacing

The use of MSCs for cartilage resurfacing of equine osteochondral defects has been investigated; however, stem cells are often coupled with a scaffold that also contributes to cartilage healing (47, 97, 98). For example, AdMSCs in fibrin glue reduced joint inflammation and improved histological and functional quality of repair tissue, but these were compared with no treatment controls resulting in significant differences despite a small sample size (97). By contrast, when compared with autologous platelet-enriched fibrin scaffold alone, addition of BMSCs did not alter biomechanical properties of cartilage at 1 year. In fact, grafts with BMSCs had increased bone edema and some horses had ectopic bone formation (98). This example highlights the need for controlling for scaffold when performing cartilage-resurfacing studies, but also highlights potential adverse effects that may occur upon differentiation of MSCs. In comparison, BMAC that contains a low number of MSC-like cells and also contains other trophic factors has been used as an alternative to culture-expanded MSCs for cartilage resurfacing (50, 54). Likely trophic and chemotactic properties improved integration, collagen, and proteoglycan content of healed tissue at 8 months compared with microfracture alone (50). Using cell-based products as anabolic and trophic stimulators may replace microfracture and improve healing by allowing continued integrity of the subchondral bone plate. Recently, Chu et al. (54) treated osteochondral defects with BMAC, finding a similar appearance to microfracture-treated defects on arthroscopy after 1 year, but there was subchondral bone fissure and void formation in the microfracture group. Resurfacing studies have also found that treatment effects occur early, or are delayed. For example, BMSC implantation in a fibrin gel glue improved histological cartilage defect healing, collagen type II, and proteoglycan content 30 days after surgery; however, there was no prolonged benefit at 8 months (47). In comparison, intra-articular scaffold-free BMSCs injected 30 days after creating an osteochondral defect and performing microfracture improved tissue repair, quality, and firmness at 6–12 m (48). The success of the second approach may be due to the trophic or anabolic effects of MSCs on already forming fibrocartilage.

### Intra-Articular Stem Cell Injection for OA

Scaffold-free intra-articular injection of MSCs has been investigated in both naturally occurring and experimental equine OA; however, the number of published clinical trials is limited compared with current commercial use Table 2. Results have been variable; which may be an indicator of the degree of inflammatory environment that varies significantly between models or naturally occurring disease, follow-up time, MSC dose and source, as well as inter-observer differences in subjective outcome parameters. Experimental *in vivo* models of equine OA and synovitis include the carpal osteochondral fragment (COF) model and the LPS-induced synovitis model with characterized levels of inflammation, cartilage matrix degradation, and lameness (99–101). The COF model mimics post-traumatic OA by creating an osteochondral fragment arthroscopically followed by exercise (102). The pathological response produces low levels of inflammation, as to be expected with naturally occurring OA (102). By contrast, acute joint inflammation can be induced by intra-articular injection of a low dose of LPS (103), which causes a transient synovitis that lasts for up to 72 h and horses recover without lasting deleterious effects (100, 103). Investigation and treatment of animals with naturally occurring OA is also a source of treatment–response information. However, there is more variation in naturally occurring OA because duration and severity of disease varies, different joints are affected, and patient signalment varies compared with a group of pre-selected experimental animals.

**Table 2:** Clinical trials using culture-expanded mesenchymal stem cells (MSCs) for OA in horses.

Disease	Stem cell type	Dose	Vehicle	Control	Results	Reference
OA—Tarso-metatarsal joint	Auto-adipose-derived MSC	5 × 10^6^	Saline	BetamethasoneNo treatment	No change in lameness at 30 days but reduced at 60 days. 180 days improvement remained in MSC group but not betamethasone group. Decreased neutrophil count at 90 days in MSC and betamethasone compared with pre-injection	Nicpoń et al. (49)
OA—Stifle, fetlock, pastern, coffin joints	Allo-peripheral blood MSCsWith or without chondrogenic induction	Not stated	Platelet-rich plasma	None	1.8% (of 165 horses) synovitis in first week, improved return to work at 18 weeks compared with 6 weeks, chondrogenic MSCs resulted in higher return to work in distal limb joints but not stifle joints	Broeckx et al. (95)
OA—due to meniscal, ligament, cartilage injury	Auto-BMSC + arthroscopy	15–20 × 10^6^	Autologous serum/5% DMSO + HA	Results compared with previous literature	Unilateral affected horses 45% return to previous work, 23% return to work, 32% failure to return to work. In comparison to previous studies without MSCs, more meniscal injuries returned to work/previous level of work. 3/33 horses had acute joint inflammation after MSC injection	Ferris et al. (33)

Models with different inflammation severity are a problem for ascertaining the treatment efficacy of MSCs because the stem cell niche induces stem cells into an anti-inflammatory phenotype. Anti-inflammatory induction of MSCs has been termed “cytokine licensing” because IFNγ and also TNFα, IL-1β, and IL-17 induce MSCs into an anti-inflammatory state (104). MSCs enhance production of anti-inflammatory and immunomodulatory factors such as TSG-6, IL-6, and PGE_2_ at higher levels of inflammation (105–110). This principle of MSC anti-inflammatory biology may explain variation between results of studies that use models with different severity of joint inflammation. The equine studies demonstrate that MSCs exposed to non-inflamed, healthy joints cause transient inflammation, evident as synovitis and increased total protein, total nucleated cell count, and inflammatory cytokines for 24–48 h (36, 58, 62, 63). By contrast, MSCs used in the most severe model for intra-articular inflammation, LPS-induced synovitis, reduced total nucleated cell count compared with LPS alone, essentially modulating the inflammatory response to LPS (63). MSC treatment has a variable effect in studies with variable or low intra-articular inflammation, as is the case with naturally occurring OA or the COF model. For example, BMSC treatment did not result in appreciable levels of reduced inflammation aside from reduction in PGE_2_ in the COF model (30). In addition, the ability of horses to return to similar athletic activities varied upon treatment of 165 horses with naturally occurring OA, which likely paralleled the variation in disease stage and joint inflammation in the population (95). Studies that investigate MSCs for OA need to be cognizant of the effect of disease stage and inflammation on the anti-inflammatory effects of MSCs.

Studies that investigate MSCs for the treatment of naturally occurring OA or articular injury in horses have limitations that need consideration before concluding about treatment efficacy. Such limitations include lack of objective outcome measures, variation in joints treated and lack of control groups. Two equine studies report improved lameness results for primary OA; however, this was a delayed response with no degree of improvement reported in one study (49), and the other lacked control groups with highly variable results and study design that included multiple different joints (95). The ability to control for specific joint analyzed is likely important given that Broeckx et al. (95) found that the 1.8% of horses that developed synovitis all had treatment of their metacarpophalangeal (fetlock) joints. Improved results have also been shown with intra-articular injection of BMSCs after stifle arthroscopy (33). However, positive outcomes may be due to the large number of concurrent intra-articular soft tissue injuries seen in the stifle, given that both equine BMSCs and AdMSCs cause healing of meniscal lesions with fibrocartilage *in vivo* (111). A cornerstone study by Murphy et al. (44) previously demonstrated that MSC-mediated meniscal and intra-articular soft tissue injury repair and subsequent return of joint stability can be a confounder in MSC studies that use instability models of OA. This can be extrapolated to the patient with naturally occurring injury. Therefore, clinical equine studies need to specify presence and degree of intra-articular soft tissue injury especially in the stifle joint. In addition, joints treated need to be defined as having or not having intra-articular soft tissue structures, for example, the palmar intercarpal ligaments in the middle carpal joints of horses and cruciate ligaments or menisci in the stifle. Overall, the functional outcomes for horses (lameness) with primary OA seem to be less consistent than those observed in other species, which may be due to the high standard of pain relief needed for horses to return to athletic use. Given the variation of efficacy found in the literature to date, further controlled studies are needed for cases of primary OA with subjective and objective functional outcome assessment to assess efficacy.

Cranial cruciate transection and/or menisectomy induce joint instability in experimental *in vivo* canine models of OA (112). As discussed, interpretation of the efficacy of MSCs is complicated in models that rely on instability to induce OA due to potential regeneration of these soft tissue structures. As a result, studies that use instability models will not be discussed. There are, however, a significant number of studies that show improved functional outcomes after treatment with AdMSCs for naturally occurring canine coxofemoral, cubital, and scapulohumeral joint OA (34, 38, 52, 53, 61, 113). The majority of clinical canine studies for primary OA are placebo-controlled, blinded, and randomized. Additionally, larger study sizes compared with equine help to account for variation in naturally occurring disease (38, 52, 53, 61). Improved functional outcome has been reported using adipose-derived stromal vascular fraction and cultured AdMSCs for the treatment of naturally occurring cubital and coxofemoral joint OA (31, 34, 38, 52, 53, 113). These therapies have shown large effect size on lameness measured by subjective grading scale, pain on manipulation and range of motion (52, 53), improved objective lameness measurements (31, 34), and overall client satisfaction with treatment (34, 38, 52, 53). A main limitation of the larger prospective, placebo-controlled studies is that objective lameness measures, such as force plate are not used (52, 53, 61). In addition, no studies compare intra-articular MSC injection to intra-articular or parenteral pharmaceuticals, which are the current standard of care in veterinary medicine. Another variable that can affect interpretation of the therapeutic efficacy of MSCs is that canine OA MSC studies vary significantly in their preparation of MSCs and the vehicle for injection ranges from hyaluronic acid, to PRP, to saline (34, 38, 52, 53, 113). Experimental studies suggest that both these factors can influence clinical outcome due to cell–vehicle interaction (32).

## Blood-Derived Products

**Figure 2:**
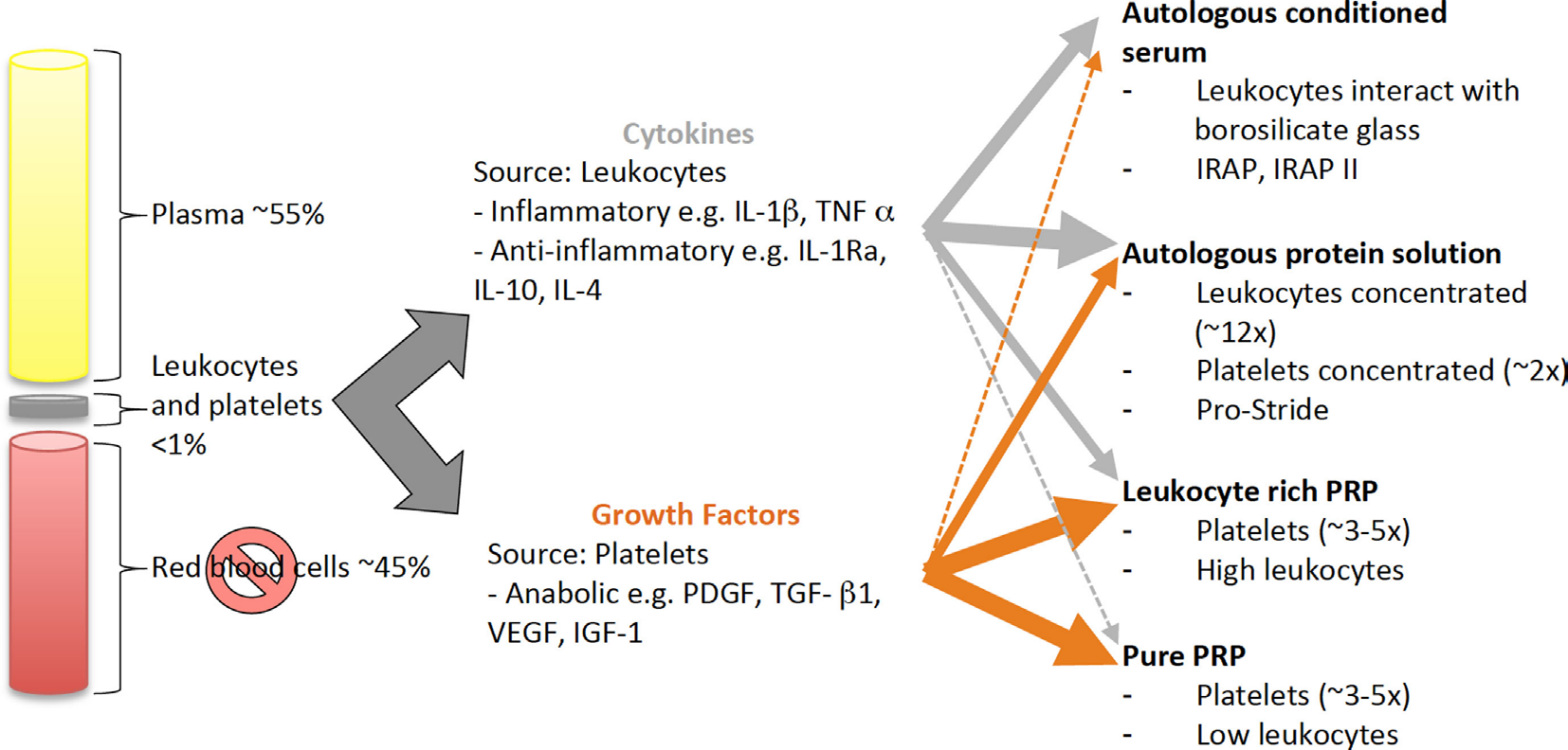
Schematic of the source and distribution of cytokines and growth factors in blood-derived cell-based products. Cytokines and growth factors are derived from leukocytes and platelets, respectively. All blood-derived cell-based products have a combination of cytokines and growth factors, which vary in amount depending on how the products are produced. Leukocytes and platelets can be concentrated by centrifugation or filtration techniques or, the cytokines and growth factors are released during the production process, e.g., by interaction with borosilicate glass and during clot formation.

Autologous blood-derived products include ACS and PRP. The concentration of platelets, presence of leukocytes and activation method, or a combination of these factors (114, 115) can further subdivide PRP products. Both ACS and PRP can be produced using kits, and defined as drugs due to the final product’s interaction with the body (116). Both ACS and PRP vary in cytokine and anabolic factor levels between and within preparation types because they are influenced by patient factors and preparation method Figure 2. These variations, as well as differing protocols used for timing and dose of intra-articular injection, make it difficult to extrapolate efficacy for the treatment of joint disease.

### Autologous-Conditioned Serum

Autologous-conditioned serum is produced by incubating whole blood with borosilicate glass beads. It was investigated as a biological treatment for OA due to increased concentration of interleukin-1 receptor antagonist (IL-1Ra), a protein that is a competitive antagonist of the main inflammatory cytokine of OA IL-1β, as well as increased presence of anti-inflammatory cytokines IL-10 and IL-4 (117). The alteration in the cytokine profile of serum is thought to be due to the interaction of leukocytes with borosilicate glass beads during incubation. ACS preparation techniques used for veterinary applications include IRAP I™ (Dechra Veterinary Products/Orthokine) and IRAP II™ (Arthrex).

Levels of anti-inflammatory and inflammatory cytokines, as well as growth factors have been investigated for IRAP I™ and IRAP II™ treatment of equine and canine blood. Both techniques increase IL-1Ra and IL-10 levels as well as growth factors IGF-1 and TGF-β1 in equine blood (16). In canine blood, IRAP I™ (20) and IRAP II™ (19) cause significantly increased IL-1ra at levels comparable to equine and human products, but no significant differences were found for other growth factors and anti-inflammatory cytokines when investigated for IRAP I™ (20). The main limitation of such studies to-date is that absolute levels, or ratios of anti-inflammatory and inflammatory cytokines have not been linked to biological or therapeutic efficacy. When equine and canine studies found that pro-inflammatory cytokines IL-1β and TNFα were also increased with ACS preparation in horses (15, 16) and dogs (20), it was suggested that the ratio of anti-inflammatory to pro-inflammatory cytokines may be important for therapeutic effect (16). However, this has not been directly shown in veterinary species. Short (<72 h) clearance time of ACS cytokines *in vivo*, coupled with minimal effects of ACS-derived TNFα on cartilage metabolism in humans may support the counter argument that cytokine profiles of ACS are of little therapeutic consequence (15). Despite lack of evidence as to the therapeutic consequence of cytokine composition, it is clear that preparation technique (15–17, 20) and individual variation (18, 19) alter the bioactive composition of equine and canine ACS. IRAP II™ has a higher IL-1Ra:IL-1 ratio than IRAP I™ in horses (16). In addition, horses that have undergone surgical stress produce ACS with reduced IL-1Ra and TGF-β1 levels that are further decreased at high levels of systemic inflammation (18); therefore, collection of blood before induction of surgical stress may be important to optimize the IL-1Ra:IL-1 ratio.

Clinical results for the treatment of equine OA with ACS have been promising; however, the precise mechanisms of action remain incompletely understood due to the action of multiple-bioactive factors in the product and few *in vitro* studies linking composition to therapeutic effect. Treatment of the COF model of equine OA with IRAP I™ injected four times at weekly intervals found decreased lameness scores, synovial thickness, and cartilage fibrillation compared with saline-treated controls to 70 days after OA induction (21). The injection frequency of ACS is likely important. Horses with arthroscopically defined naturally occurring OA treated with three injections of IRAP II™ at 2-day intervals had significantly lower levels of IL-1β, biomarkers of cartilage degradation, and IL-1Ra 42 days after treatment initiation compared with horses injected at 7-day intervals (22). Despite clinical improvements, *in vitro* studies have not demonstrated chondroprotective effects and it is likely that mitigation of inflammation is mainly responsible for reduction in lameness and cartilage degradation. Although ACS (IRAP II™) increased IL-1Ra and IGF-1 in equine cartilage explants treated with IL-1β, there was no significant difference in MMP-3 production and proteoglycan loss or synthesis between ACS and serum-treated samples suggesting minimal beneficial effects of ACS on cartilage matrix metabolism (17). Taken together, these results suggest that ACS predominantly acts as a mild anti-inflammatory agent in the joint. While the benefits of reduced inflammation during OA are clear, the benefit of ACS over pharmacological anti-inflammatories such as triamcinolone acetate are not because preclinical and clinical veterinary studies have not included positive control corticosteroid groups. In addition, the effects on articular cartilage are unlikely sufficient to support DMOAD effects at this time.

A variant of ACS called APS, Pro-Stride™ (Biomet Biologics), has been gaining clinical popularity because the product does not require an incubation period and has been investigated using a single intra-articular injection in horses and dogs. A bench-top centrifuge firstly isolates white blood cells, platelets, and plasma proteins, then they are further concentrated in a second centrifugation step (29). Pro-Stride™-treated equine blood resulted in a leukocyte count 12 times and platelet count 1.6 times higher than whole blood (29). The increased leukocytes result in elevation of anti-inflammatory cytokines such as IL-1Ra, IL-10, and soluble TNF receptor 1 (29). The same study compared one intra-articular injection of Pro-Stride™ to saline control in horses with naturally occurring OA. The APS group had significantly more horses that were sound or had improved by approximately one AAEP lameness grade at 7 and 14 days. However, such favorable outcomes occurred in horses with no radiographic signs of moderate-to-severe osteophytes, subchondral sclerosis, or joint space narrowing (29). The advantage of this study was that it was performed in horses with naturally occurring OA; however, the follow-up period for objective lameness and biochemical data was short (14 days) and joint type varied. In addition, outcome was significantly linked to stage of OA and a non-significant trend for the APS group to have lower radiographic evidence of disease and reduced synovial inflammation pre-injection, may have influenced outcome. Wanstrath et al. (28) also demonstrated a positive effect on canine lameness and pain scores with a single Pro-Stride™ injection compared with saline-treated controls. Both studies exhibited transient synovitis in the initial period after injection, which is likely due to the high leukocyte content of the products. High leukocyte content increases inflammatory cytokine content for other biologics such as PRP (118). However, in both studies, the levels of inflammatory cytokines such as IL-1β and TNFα in the product were not investigated. In addition, effects on cartilage matrix metabolism are unknown so DMOAD effects cannot be claimed. Further studies that compare APS to traditional pharmacologic drugs and indentify effects on disease progression are needed before widespread use and disease-modifying claims for APS.

Although there are relatively few studies in horses and dogs regarding intra-articular use of ACS and APS, they all show evidence of mild symptom or inflammation-relieving effects. The promise to additionally provide regenerative or disease-modifying effects is yet to be realized and their potency compared with traditional symptom-modifying OA drugs, such as corticosteroids, has not been investigated. However, predominantly in equine sports medicine, clinicians are faced with insulin resistant patients, those with previous laminitic episodes, or competition medication rules that prevent them using intra-articular corticosteroids (11–13). Autologous anti-inflammatory biologics have a niche to treat such patients and, as a result, will continue to have clinical utility. Major areas of investigation that lag behind clinical misconception and opinion are their efficacy to have disease-modifying effects, the relevance of biological composition, comparison to traditional pharmacological anti-inflammatory drugs, and their ability to have long-term intra-articular presence or therapeutic effects.

### Platelet-Rich Plasma

Platelet-rich plasma is the plasma portion of the patient’s own blood that has an increased concentration of platelets through centrifugation or filtration steps. Alpha granules in the concentrated platelets are the source of both growth factors and cytokines. They release primarily PDGF and TGF-β1, but also VEGF and IGF-1 (119) when they are activated by the disease environment, before injection through the use of CaCl_2_, thrombin, or a combination, or platelets are lysed during freeze–thaw cycles. It must be noted that PRP is perhaps the most variable of the blood-derived cell-based products because it has been shown to vary in the number of platelets, white blood cells, activation technique, and fibrin content depending on what preparation technique is used Table 3 (114, 115, 120, 121). Furthermore, within PRP types and individuals, the concentration of platelets and leukocytes can vary. For example, horses given NSAIDs had increased platelet concentrations and the leukocyte concentration was elevated by dehydration and sampling at night (122).

**Table 3:** Average platelet and leukocyte counts reported for commercially available platelet-rich plasma systems in the horse.

	Platelets/μL (fold Δ)^a^	Leukocyte/μL (fold Δ)^a^	Reference
Pall corporation	542,000 (3.2)	13,000 (1.9)	Textor and Tablin (123)
E-Pet/V-pet^b^	533,300 (3.8)	11,000 (1.8)	Hessel et al. (121)
	550,000 (~4)	–	Mirza et al. (24)
Harvest	513,000 (5.54)	6,910 (NC)	McCarrel and Fortier (124)
SmartPrep2	725,000 (4.2)	14,800 (~2)	Kisiday et al. (125)
Arthrex ACP	276,000 (1.6)	30 (~0.005)	Kisiday et al. (125)
	183,000 (1.3)	600 (0.1)	Hessel et al. (121)
Arthrex Angel	320,300 (2.3)	9,100 (1.5)	Hessel et al. (121)
Biomet GPS III	761,000 (5.4)	40,600 (6.7)	Hessel et al. (121)

*^a^Fold Δ is over whole equine blood, NC = no change, – represents data not available*.

*^b^Final platelet diluent in E-Pet/V-Pet system is hypertonic saline, not plasma*.

There is growing controversy over how concentrated platelets need to be for positive therapeutic effect. In some cases, optimum platelet concentrations are quoted in product manufacturer websites with no stated reference, which disregards the differences in platelet activity between species or over-represents the understanding we have of these products in horses and dogs (126). Both the structure and mechanism of degranulation differ between human and equine platelets, making it difficult to draw direct comparisons between species (124, 127). The minimum platelet concentration that defines human PRP is >1 million platelets per μL (128), which is approximately two to six times more concentrated than whole blood. PRP is occasionally referred to as autologous platelet concentrate; however, this should be reserved for platelets that are maximally concentrated rather than increased above baseline. There are no minimum platelet concentrations or fold increase over systemic platelet count defined for equine or canine PRP. While there are no studies currently investigating the effect of platelet concentration on therapeutic efficacy for OA, horses that had tendinopathy treated with >750,000 platelets per μL (approximately five times baseline), returned to work in 3 months compared with 8 months for those treated with less concentrated PRP (129). However, more is not better in regard to platelet concentration. Boswell et al. (118) found an apparent concentration/benefit plateau where tendon metabolism decreased at high platelet concentrations in a linear manner, although the specific platelet count at the plateau point was not defined. Despite controversy over the exact fold increase in platelet count, specific guidelines for equine or canine PRP have not been set and may differ between tendinopathy and OA.

Available systems to make PRP for horses and dogs concentrate platelets to varying degrees, which influences growth factor levels (120, 121). Growth factor levels are directly correlated to platelet concentration in horses and dogs; however, it is unclear how growth factor levels influence OA or cartilage metabolism in these species (118, 120, 121). Human studies show that TGF-β1 and IGF-1 stimulate extracellular matrix synthesis from chondrocytes (130, 131) and IGF-1 decreases synovial inflammation (132). However, high physiological levels of TGF-β1 have undesirable effects on the synovium in mice including increased leukocyte infiltration, synovial fibrosis, and osteophyte formation (133). It is unclear if these effects are related to a high concentration of a single growth factor, and if the growth factor mileu in PRP would cause similar results. Platelet-derived growth factor stimulation of human synoviocytes causes production of hyaluronic acid, which may be a source of indirect anti-inflammatory activity and enhance joint lubrication (134). It is likely that PRP’s mechanism of pain modulation or anti-inflammatory activity is multimodal and its efficacy could be related to the stage of OA. No differences were found between people treated with PRP or hyaluronic acid (135) unless cartilage degeneration was present, where there was a trend for improved pain and motion in the pure PRP (PPRP) group vs. the hyaluronic acid group (135, 136). Optimum platelet concentration and the effects of growth factor levels in equine and canine OA are currently unknown, leaving us to extrapolate from human or species-specific tendon research. This approach is less than ideal given both species- and disease-specific differences.

Perhaps the most important source of variation when considering PRP for intra-articular use is the leukocyte content, which has been related to the degree of catabolic signaling induced by collagen matrix in horses (118). Liquid-phase PRP used for intra-articular injection in veterinary patients can be defined as PPRP, which is leukocyte-reduced over whole blood, or leukocyte and platelet-rich plasma (LPRP). PPRP is termed as such to denote a more uniform (“pure”) presence of platelets vs. other cellular components; however, it is impossible for all leukocytes to be removed during PRP processing so the term “leukocyte-reduced PRP” is occasionally, and more correctly, used to describe this PRP subtype. A potential limitation of PRP is that inflammatory cytokines including IL-1β, IL-6, and IL-8 have been found using different preparation techniques with human blood (137, 138). Inflammatory cytokines are related to leukocyte content and can be reduced by leukocyte depletion (138). Both leukocyte content and PRP concentration are likely important for the biological effect on synovium, cartilage, and meniscus (125, 139, 140). IL-1ra was increased in LPRP compared with PPRP, and LPRP used at low concentrations as a gel (which had the confounding effect of reducing LPRP concentration) had the greatest anti-inflammatory and anabolic effects on synovial and cartilage explants (139, 140). However, increased to maximal concentration of leukocytes may have a detrimental effect on both cartilage and meniscal metabolism (125), so LPRP should be used cautiously in joints and if so, used at low concentrations, which are yet to be defined.

Pure PRP use for equine OA has shown some success in clinical studies; however, compared with ACS, there are less controlled studies, study sizes are extremely small, and production and activation techniques of PPRP offer inherent variation. PPRP improved lameness and effusion scores in a pilot study of 4 horses that was maximal 2 months after injection and persisted for 8 months (23), and PPRP with lysis of platelets *via* freeze–thaw improved lameness associated with distal interphalangeal joint OA compared with a saline control in 10 horses (25). In addition to the small number of horses, there was either little or incomplete analysis of the growth factor and cytokine profiles of the products tested. There is high variability associated with preparation system (139), platelet activation (141), and individual horse factors (119) that could affect the clinical response to PPRP treatment in horses and require a greater study size to achieve results that can extrapolate to larger populations. As discussed above, both platelet and leukocyte content vary the growth factor and cytokine content of PRP and could affect therapeutic efficacy.

Perhaps the largest source of variation in therapeutic response and largest concern for safety lies with activation method. PRP is most commonly administered in its non-activated state; however, activation *via* bovine thrombin or calcium chloride can be used in an attempt to enhance degranulation of platelets and subsequently growth factor release. When bovine thrombin-activated PPRP was injected into healthy metacarpo-/metatarsophalangeal joints in horses, there were higher levels of growth factors released, but it caused joint effusion and generalized distal limb soft tissue swelling (123) with increased synovial fluid TNFα and IL-6 (141). The authors guarded against the safety of bovine thrombin due to the apparent inflammatory reaction to this xenogeneic protein. They recommended the use of non-activated or calcium chloride-activated PRP for intra-articular use, which had no adverse reactions. No controlled clinical investigation of PPRP for OA treatment in horses has been performed, and so-far clinical improvement after injection is variable (24, 25). In addition, a positive response to intra-articular anesthesia does not ensure reduction of lameness after PPRP injection (24). Further research to ascertain the efficacy of PPRP products derived from various systems needs to be performed before widespread use for OA.

In contrast to ACS, more research has been performed for the intra-articular use of PRP clinically in dogs. In dogs with OA, a single intra-articular LPRP treatment (3-fold increase in platelet count, 1.8-fold increase in leukocytes) decreased objective and subjective lameness and comfort scores compared with baseline or placebo controls (26), and pain-relieving effects were not significantly different from traditional intra-articular therapy of corticosteroid and hyaluronic acid (27). It must be noted that in these studies the leukocyte count was significantly higher (1.8 times systemic), constituting LPRP (26), or the leukocyte and platelet counts were not reported (27). The difference between PRP and traditional therapy demonstrated in dogs is that maximum pain-relieving response is seen at approximately 1 week with traditional therapy, but is most prominent after 6 weeks with PRP therapy (27). A slow onset of maximum therapeutic response was also seen when PRP was combined with AdMSCs (34) and was observed in a small number of horses (23). In these studies, PRP was used in a non-activated state. The lag in therapeutic response may be due to the gradual release of growth factors when platelets are allowed to be activated by the disease environment, shown experimentally over 4 days in an equine tendon explant model (124) and over 9 days in healthy equine joints (123). Experimental canine models using PRP suggest that reduction in synovitis as well as reduced collagen break down and matrix metalloproteinase activity could be responsible for the positive therapeutic response (142, 143). If similar disease-modifying effects could be shown in naturally occurring OA, PRP may reach DMOAD status in the future. However, PRP will need to overcome significant challenges associated with the variation discussed to prove that it is consistent and effective for the treatment of OA in veterinary species.

### Summary

It is clear that both blood-derived cell-based products and MSCs have a complicated pathway from harvest to the end-user with scope for variations that make cell-based products different even within the same category. Variation transfers to the patient and is compounded for MSCs because they react to the specific disease environment encountered, making results between and within studies variable. The variation between veterinary cell-based studies could allow researchers to determine favorable protocols, but will not allow consistent and safe cell-based products to be produced. If veterinarians want effective, consistent cell-based products, research needs to be shared and the quality adequate to select protocols that allow similar production techniques, shipping and injection methods, and standardized outcome parameters. This approach would allow some uniformity between studies and allow meta-data analysis to produce meaningful conclusions. As the variation of veterinary cell-based research stands, synthesis of study results to draw meaningful conclusions is difficult, if not impossible. Regulatory bodies recognize variation in veterinary cell-based products and the potential risk to our patients. The FDA has been a world-leader in publication of guidelines that aim to get researchers and clinicians recording their protocols, quality control measures, and treatment results. Using the regulatory pathways that make other drugs safe and effective is certainly the preferred pathway for veterinary cell-based products to take from a clinical standpoint. From a biological standpoint, inherent variation in source and donor will make regulatory pathways challenging. Therefore, a solid understanding of underlying cell biology is imperative for researchers, clinicians, and regulatory agents.

## Regulatory Aspects of Cell-Based Therapies

Regulatory and ethical aspects of stem cell therapy are topics of global discussion. However, even for human stem cell research and treatment, regulatory control varies internationally, from creation of national stem cell banks and regenerative medicine select committees in Britain, to minimal regulation or forum for data collection in other countries. Overall, most European countries, as well as Asia-Pacific have some bioethics legislation for the use and acquirement of human stem cells (144). In contrast, there is little regulation for the collection or use of animal stem cells for research or clinical purposes. In the USA, the governing body for both human and animal food and drugs is the FDA. Specifically, the FDA’s Center for Veterinary Medicine (CVM), controls approval of animal drugs. The CVM has been the only legislative body to-date to formally publish specific definitions and recommendations for veterinary cell-based products, in a direct response to the growing use of cell-based products in animals clinically (76, 77). The guidance not only foreshadows where enforceable legislation will lead but is also an example of how other regulatory bodies may provide a framework for researchers and clinicians to record manufacturing processes and clinical results. These steps are expected to promote achievement of product consistency, safety, and efficacy to enhance the welfare of our patients.

### How Does the FDA Regulate Cell-Based Products?

Stem cell-based products, as well as those derived from whole blood like ACS and PRP are defined by the FDA as “cell-based products” as they contain, consist of, or are derived from cells. There are many types of cell-based products currently being marketed to the veterinary industry including stem cells, ACS, PRP, and in-clinic kits used to produce these products. FDA guidelines define cell-based products as an animal drug because they are intended for use in the diagnosis, cure, mitigation, treatment, or prevention of disease and are articles intended to affect the structure or any function of the body [21 U.S.C. 321 Section 201(g)(1)(B) & (C)]. FDA guidance states that manufacturers of cell-based products meeting the definition of a new animal drug are subject to the same statutory and regulatory requirements as manufacturers of other new animal drugs. Therefore, cell-based products are required to go through pre-market review of experimental data to ensure that the product is safe, effective, and high quality before marketing of the product (145, 146). Currently, the FDA does not define specific *in vitro* or *in vivo* models that are needed or accepted for experimental data on veterinary species. Rather, the focus for regulation is on clinical trials using client-owned animals with naturally occurring disease. This process is regulated by the FDA’s CVM, who published guidance regarding the regulation of cell-based products for animal use in 2015 (76, 77). The FDA recommends directly contacting them if researchers are considering pre-market review of experimental data, which may be done before data collection to ensure adequately detailed results.

There are currently no animal cell-based products that are FDA approved and can be legally marketed (145, 146).

### How Do FDA Guidelines Affect Cell-Based Therapies?

As discussed, MSCs have been used for the treatment of intra-articular soft tissue injury (33, 45) and cartilage regeneration (47, 48) in veterinary species. In June 2015, the FDA released guidelines for the veterinary industry on cell-based products (76, 77). The guidelines defined and categorized types of cell therapies to clarify what products require an approved New Animal Drug Application (NADA) before legal marketing. The guidelines classified products from other species (xenogeneic), other individuals of the same species (allogeneic), and those from the same individual (autologous). Autologous cell therapies were divided into two categories: type I and type II. Type I are autologous cell-based therapies that are more than minimally manipulated (have processing that alters their relevant biological characteristics, such as expansion, addition, or purification of a cell-based factor); intended for non-homologous use (replacement of recipient tissue with a cell or tissue that does not perform the same basic function in the recipient as it did in the donor); intended for use in a food producing animal; dependent on the metabolic activity of its living cells for effect; or combined with other articles, drugs, or devices. Examples of autologous type I include any stem cells expanded in culture or cell-based products derived from fat or bone marrow used for cartilage repair. By contrast, type II autologous cells are minimally manipulated (for example, centrifugation); intended for homologous use; intended for use in non-food producing animals; and are not combined with other articles, drugs, or devices. An example of type II would be isolated non-expanded chondrocytes used to fill an articular cartilage defect.

### The Pre-Market Review Process

If an investigator, manufacturer or practitioner has a xenogeneic, allogeneic, or type I autologous cell-based therapy that they intend to market or investigate in client-owned animals, it is recommended that they contact FDA to discuss the appropriate pathway for their product. The suggested route for pre-market approval of cell-based products is through the NADA pathway. The requirements for approval of a NADA include, in part, demonstration of safety, effectiveness, and manufacturing quality (76, 77). The regulations also provide a pathway for investigational use allowing for the conduct of research to gather information necessary to demonstrate safety and effectiveness. Investigational use of cell-based products in client-owned animals may be conducted under a clinical investigational exemption. The clinical investigational exemption contains a number of conditions including items such as prior notice of shipment, or delivery of the investigational product, and reporting of study information and adverse events to an Investigational New Animal Drug (INAD) file. The investigational exemption also prohibits marketing or commercializing the investigational product. Investigational use of cell-based products intended solely for *in vitro* studies or laboratory research animals (non-client-owned animals) may be conducted without establishing an INAD file.

It is recommended to contact the FDA’s CVM if you are currently manufacturing or intending to manufacture or use a cell-based product for use in client-owned animals so that the correct steps are taken. An explanation of how the guidelines could impact institutions currently marketing and manufacturing cell-based products, as well as contact information for the FDA’s CVM, can be found in the FDA’s letter to veterinary schools (76, 77). Currently, no FDA involvement is needed for use of these products solely in research or laboratory animals (animals that are not client-owned).

### Cell-Based Products and the Regulatory Future

The regulatory standards for autologous cell and tissue-derived products in veterinary medicine are now a reality for investigators, manufacturers, and sole practitioners in the USA. It is likely that tighter regulatory standards will spread globally. Although creating a record with a regulatory body, like an INAD file, may seem like an administrative burden, the process has key benefits to the industry by obligating us to collect and record data on our patients. This opportunity to combine data on multiple patients treated with experimental cell-based products will likely secure a future for safe, effective products in our veterinary species. Since the FDA’s 2015 guidelines, both academia and industry in the USA have moved toward involving the FDA to ensure a head-start in the future regulatory and competitive environment. Compared to pharmaceuticals, cell-based products have inherent variation as the levels of cytokines, growth factors, stem cell activity, or other biological response modifiers vary with multiple factors that include individual, diurnal variation, environmental stress, and processing procedures (16, 18, 147–149). Therefore, manufacturers will be faced with the challenge of proving that a product’s strength, quality, and purity are maintained from batch to batch to demonstrate efficacy. Overcoming this limitation of cell-based products will require a solid understanding of the cellular and molecular biology behind the manufacture and use of cell-based products, as well as ensuring that regulatory reviewers understand the inherent variation. Regulators, scientists, and industry will need to work together to understand the critical parameters impacting the safety and effectiveness of these products, and to set appropriate standards for approval of cell-based products.

## Conclusion and Perspective

Review of the veterinary cell-based literature to-date emphasizes that as we learn more from published findings, the scope for variation within and between cell-based therapies grows. Effectively, what we have learned is that it is impossible to draw finite conclusions from current data. The difficulties are inherent to the field because variation can occur during multiple stages from harvest to therapeutic effect, such as the source, manufacturing processes, shipping techniques, administration techniques, and disease environment. In addition, variation is complex within the formulation of each cell-based product because of the multiple-bioactive factors that can be affected, and the influence that different components may have on each other and the joint environment. Examples described in this review included PRP, where the amount of platelets and leukocytes may affect how the product performs in an intra-articular environment, or MSCs that can be affected by the degree of inflammation during OA and synovitis. Even investigation of levels of bioactive components can seem futile when it is unclear what components have the largest influence on therapeutic response. Deciphering true therapeutic response from clinical variation is complicated by studies that have low numbers, use different joints, stages of disease, vary between experimental and naturally occurring OA, or have different outcome parameters. Unfortunately, these issues are inherent to veterinary research due to reduced opportunities for funding and the high expense of cell-based as well as large-animal research.

Proving safety, consistency, and effectiveness of cell-based products is the best way to protect our patients and ensure longevity of the field. It is clear that the current variable approach to preclinical and clinical research does not allow clear conclusions about any of these essential facets of our cell-based therapeutics. Regulatory agencies like the FDA have recognized this and are influencing veterinary cell-based product manufacturers to pre-plan and record data in an attempt to standardize clinical research. However, only a fraction of veterinarians and researchers using or investigating cell-based products will be influenced by regulation. To move the field of veterinary cell-based therapies forward, the solution is less about what we need to know and more about what we need to do. Practitioners and researchers must collaborate globally, mimicking a regulatory body, if safe, effective, and consistent cell-based products are desired.

**Figure 3:**
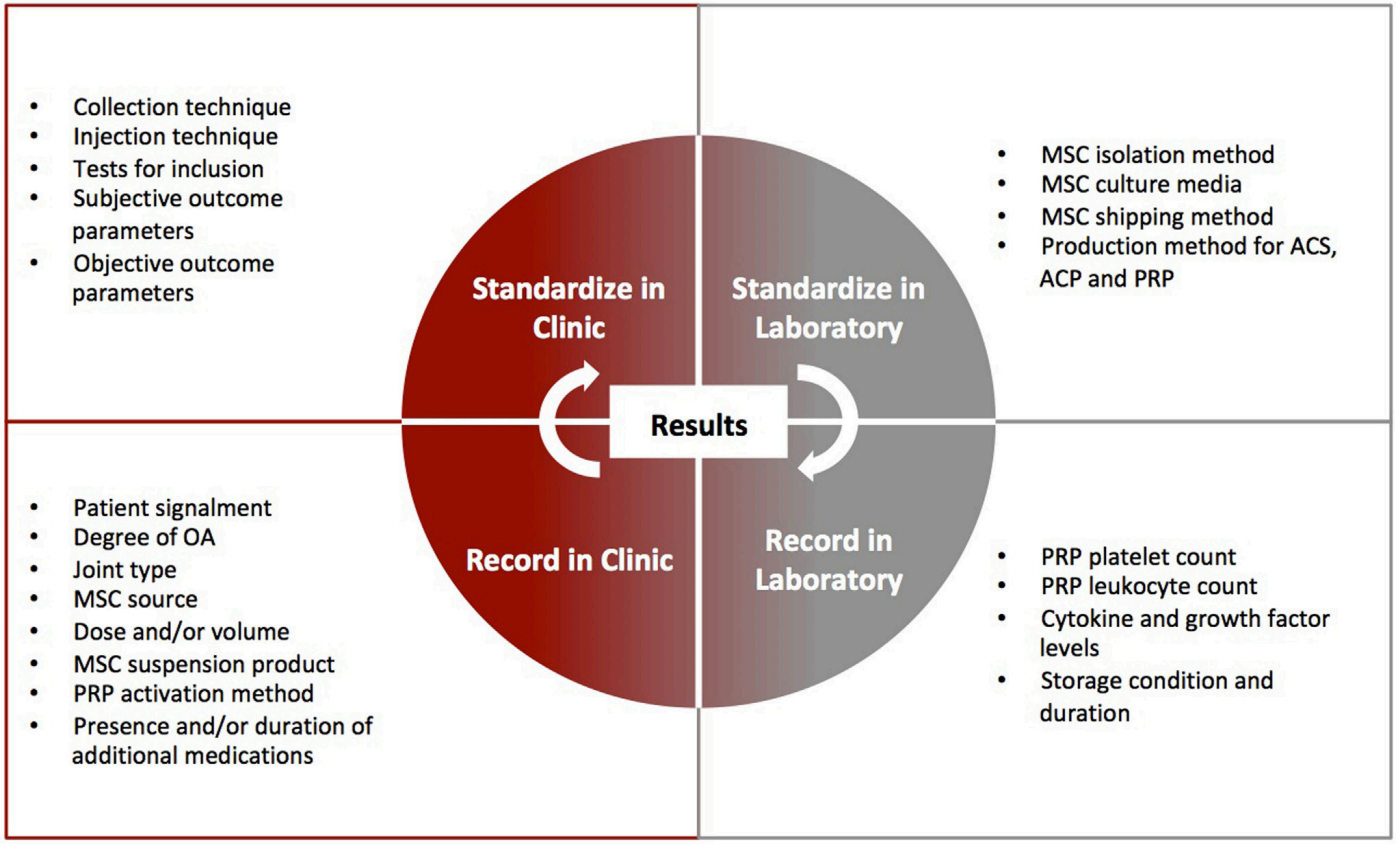
Outline of clinical and laboratory parameters that can be standardized or recorded to enhance interpretation of clinical trial results for cell-based therapy. Standardization and recording for cell-based therapies will be imperative in regulatory approval pathways.

Through already formed professional bodies, veterinarians and veterinary researchers need to create a cell-based therapy forum. Ideally, action is needed to create uniformity between studies that include standardized preparation methods and transport conditions for MSCs, enforced reporting of platelet and leukocyte composition and activation technique in PRP, resolution of what inflammatory and anti-inflammatory cytokines are important to report for ACS or ACP, MSC studies with standardized cell culture media and suspension product, analysis by joint type and disease stage in clinical studies, performance of dose–response studies, appropriate and standardized experimental models in the absence of naturally occurring OA and recommend a standardized set of outcome parameters for clinical trials within each species Figure 3. Such uniformity will enable more direct comparisons between studies, as well as pooling of data for meta-analysis so that we can draw conclusions about symptomatic and disease-modifying effectiveness of cell-based therapies, which is what we need to know. A counter argument is that the variability seen so-far has allowed discovery of novel approaches to cell-based therapy, and that the expense of veterinary studies contrasts to reduced animal-specific funding compared with human medicine. However, variability is a direct challenge for cell-based products in their pathway to become safe, effective therapeutics. Unless we can prove consistency, cell-based products may not endure regulatory processes. A practical solution to funding constraints is for practitioners to form alliances with veterinary researchers. This will allow them to treat patients with cell-based therapies in a pre-defined manner and record outcomes that can be analyzed rather than the current trend, whereby many treatments on client-owned animals are not recorded as part of research. In the USA, the FDA has encouraged record keeping in the form of an INAD file. Globally, professional bodies could provide a forum for collaboration as well as provide access for recording and analysis of results. As our collaboration and understanding of the effect of cell-based therapies on OA improves, so too will the transition of cell-based therapies from variable but promising therapeutics to consistent and effective drugs for OA.

## Author Contributions

This author performed all literature review, writing, and editing of this article.

## Conflict of Interest Statement

The author declares that the research was conducted in the absence of any commercial or financial relationships that could be construed as a potential conflict of interest.
